# Responses of Woody Plant Functional Traits to Nitrogen Addition: A Meta-Analysis of Leaf Economics, Gas Exchange, and Hydraulic Traits

**DOI:** 10.3389/fpls.2018.00683

**Published:** 2018-05-23

**Authors:** Hongxia Zhang, Weibin Li, Henry D. Adams, Anzhi Wang, Jiabing Wu, Changjie Jin, Dexin Guan, Fenghui Yuan

**Affiliations:** ^1^Key Laboratory of Forest Ecology and Management, Institute of Applied Ecology, Chinese Academy of Sciences, Shenyang, China; ^2^University of Chinese Academy of Sciences, Beijing, China; ^3^State Key Laboratory of Grassland and Agro-Ecosystems, Key Laboratory of Grassland Livestock Industry Innovation, Ministry of Agriculture and Rural Affairs, College of Pastoral Agriculture Science and Technology, Lanzhou University, Lanzhou, China; ^4^Department of Plant Biology, Ecology and Evolution, Oklahoma State University, Stillwater, OK, United States

**Keywords:** atmospheric nitrogen deposition, hydraulic conductance, intrinsic water-use efficiency, leaf area index, leaf economic traits, meta-analysis

## Abstract

Atmospheric nitrogen (N) deposition has been found to significantly affect plant growth and physiological performance in terrestrial ecosystems. Many individual studies have investigated how N addition influences plant functional traits, however these investigations have usually been limited to a single species, and thereby do not allow derivation of general patterns or underlying mechanisms. We synthesized data from 56 papers and conducted a meta-analysis to assess the general responses of 15 variables related to leaf economics, gas exchange, and hydraulic traits to N addition among 61 woody plant species, primarily from temperate and subtropical regions. Results showed that under N addition, leaf area index (+10.3%), foliar N content (+7.3%), intrinsic water-use efficiency (+3.1%) and net photosynthetic rate (+16.1%) significantly increased, while specific leaf area, stomatal conductance, and transpiration rate did not change. For plant hydraulics, N addition significantly increased vessel diameter (+7.0%), hydraulic conductance in stems/shoots (+6.7%), and water potential corresponding to 50% loss of hydraulic conductivity (*P*_50_, +21.5%; i.e., *P*_50_ became less negative), while water potential in leaves (−6.7%) decreased (became more negative). N addition had little effect on vessel density, hydraulic conductance in leaves and roots, or water potential in stems/shoots. N addition had greater effects on gymnosperms than angiosperms and ammonium nitrate fertilization had larger effects than fertilization with urea, and high levels of N addition affected more traits than low levels. Our results demonstrate that N addition has coupled effects on both carbon and water dynamics of woody plants. Increased leaf N, likely fixed in photosynthetic enzymes and pigments leads to higher photosynthesis and water use efficiency, which may increase leaf growth, as reflected in LAI results. These changes appear to have downstream effects on hydraulic function through increases in vessel diameter, which leads to higher hydraulic conductance, but lower water potential and increased vulnerability to embolism. Overall, our results suggest that N addition will shift plant function along a tradeoff between C and hydraulic economies by enhancing C uptake while simultaneously increasing the risk of hydraulic dysfunction.

## Introduction

Atmospheric N deposition has increased by three-to five-fold over the past century due to increased fossil fuel combustion and artificial fertilizer application (IPCC, 2007; Davidson, 2009) and has been predicted to increase by as much as twice the current level by 2050 (Galloway et al., 2008). Atmospheric N deposition has many negative effects on terrestrial ecosystems, such as soil acidification, nitrate leaching and the loss of biodiversity (Hoegberg et al., 2006; Clark and Tilman, 2008; Dise et al., 2009; De Schrijver et al., 2011). However, increased nitrogen deposition can also stimulate plant growth and terrestrial ecosystem production due to higher N availability (Reay et al., 2008; Thomas et al., 2010; Li et al., 2015). Increased plant growth with increasing N availability brings changes in plant leaf economics, gas exchange (Hacke et al., 2010), and hydraulic architecture (Borghetti et al., 2017), which in turn impact plant water relations (Kleiner et al., 1992; Goldstein et al., 2013). Changes in leaf economics, gas exchange, and hydraulic traits of woody plants may influence plant survival and resistance under environmental stresses, particularly during drought. On the other hand, hydraulic architecture and water relations strongly influence allometric scaling in the size, form, and population density of woody plants, and hence impact plant growth and the terrestrial ecosystem C sink (Savage et al., 2010; Blonder and Enquist, 2014). Therefore, through understanding the effect of long-term N addition on woody plant leaf economics, gas exchange and hydraulics we can better understand changes in plant growth and terrestrial productivity.

Xylem structure is plastic in response to climate change, and most N-addition experiments revealed that higher N availability increased vessel diameter (Watanabe et al., 2008; Plavcová and Hacke, 2012), and increased xylem conductance for water transport (Bucci et al., 2006; Goldstein et al., 2013), which likely enhances xylem efficiency (Hacke et al., 2010), but it is not clear if this is a general pattern. Higher hydraulic efficiency of water transport in xylem structure in woody plants concurrently increases the risk of cavitation and stress-induced embolism (Harvey and Van Den Driessche, 1999; McElrone et al., 2004; Wheeler et al., 2005; Villar-Salvador et al., 2013; but see Gleason et al., 2016). On the other hand, a meta-analysis revealed that vessel density increased with N deposition, which may improve capacity to withstand the risks of drought-induced embolism, thereby preserving xylem safety (Borghetti et al., 2017). Hence, these changes add uncertainties to the effects of N deposition on plant hydraulics.

The changes in xylem structure with increased N deposition are attributed to the stimulation of radial growth due to increased photosynthetic assimilation that results from enhanced plant foliar N content (Evans, 1989; Grassi et al., 2002; Camarero et al., 2017). Besides these effects on wood anatomical traits, increased carbohydrates produced from photosynthesis may be invested in increased leaf production and cause changes in leaf morphology. Resulting increases in total leaf area can reduce leaf-specific hydraulic conductivity and minimum leaf water potentials while concurrently increasing plant water loss (Bucci et al., 2006). In addition, concentrations of stored carbohydrates, in form of soluble sugars, in plant organs (e.g., leaves, roots) can influence osmotic pressure and water potential. Because soil N availability also influences C allocation patterns, variations in biomass production and concentrations of stored carbohydrates among leaf, stem and root can affect the hydraulic structure and water potential among different organs in individual plants (McCarthy et al., 2006). Moreover, stomatal conductance plays a critical role in the intrinsic water-use efficiency (*iWUE*) trade-off between C gain and water loss at the leaf level. Plants can regulate stomatal opening to maintain xylem water potential under variable environmental conditions, which in turn affects hydraulic conductance and *iWUE* (Sperry et al., 2002; Vilagrosa et al., 2003; Cochard et al., 2004). Results of a previous synthesis emphasized the importance of N availability on *iWUE* for crops (Brueck, 2008).

To date, global observations of altered woody plant leaf economics, gas exchange, and hydraulics have inspired a thrust of studies on effects of N addition (Jennings et al., 2016; Wang et al., 2016). However, such studies have often focused on a single species in a single experiment, and thereby do not allow us to derive general patterns. Although studies have been conducted in multiple plant species, the variability of results among different plant taxa remains unclear. Moreover, different types of N fertilizers (e.g. NH_4_NO_3_, Urea, ${\text{NH}}_{4}^{+}$-N and ${\text{NO}}_{3}^{-}$-N) were applied, which further complicates comparison (Ruan et al., 2000). N-addition levels have ranged from several kg N ha^−1^ yr^−1^ to hundreds kg N ha^−1^ yr^−1^, and the duration of N treatment has also varied from several weeks to a dozen years. Hence, without synthesis across these studies, the general responses of plant leaf economics, gas exchange and hydraulics to increasing atmospheric N deposition are not well characterized.

Here, we compiled 631 observations from 56 experimental studies and conducted a meta-analysis to identify the general pattern of the effects of N addition on woody plant leaf economics (specific leaf area, leaf area index and foliar N content), gas exchange (stomatal conductance, transpiration rate, net photosynthetic rate and *iWUE*) and hydraulic traits (hydraulic conductance, water potentials, water potential corresponding to 50% loss of hydraulic conductivity (*P*_50_), vessel density and vessel diameter). Our objective was to investigate the general patterns in N addition effects on leaf and xylem traits of woody plants and the differences in response among plant taxa and settings of N addition experiments (N fertilization types, N-addition levels and treatment duration).

## Materials and methods

### Data collection

We collected data from 56 peer-reviewed journal articles (Supporting Information, Data Sheet 1) published from 1990 to 2017 by using the Web of Science resource. The search terms were “nitrogen addition” or “fertilization” or “nitrogen deposition” combining with “hydraulic” or “stomatal conductance” or “water use efficiency” or “physiology.” A total 631 observations of 15 variables related to foliar economics, gas exchange and hydraulic traits were identified. Our criteria were as follows: (1) Only studies on woody plants were included (e.g., herbaceous plants and crops were excluded); (2) Only field simulated nitrogen deposition studies were selected and laboratory incubation studies were excluded; (3) At least one of the selected variables was measured directly (e.g., model simulated values were excluded); (4) Only control and nitrogen addition treatment data were selected and results from interacting effects in multifactor experiments were excluded; (5) Fertilizers used contained only nitrogen and no other nutrient (e.g., K, P, and Ca); (6) The means, standard deviations (SD) or standard errors (SE), and sample sizes (n) were directly provided or could be calculated from the studies. Data were compiled from Tables or extracted from Figures of the published articles using Engauge software (4.1). Studies with multiple plant species or canopy height were treated as separate entries; when variables were reported for multiple sampling dates, only the monthly means (individual value or means from multiple values during each calendar month) were analyzed.

### Categorization of studies

In this meta-analysis, the selected 15 variables were categorized into two groups: (1) Leaf economics and gas exchange (including specific leaf area, leaf area index, foliar N content, stomatal conductance, transpiration rate, net photosynthetic rate and *iWUE*); (2) Hydraulics [including hydraulic conductance in leaves, roots and stems/shoots, water potential in leaves and stems/shoots, water potential corresponding to 50% loss of hydraulic conductivity (*P*_50_), vessel density and vessel diameter].

For each study, we noted plant taxonomic groups, N fertilizer types, N-addition levels and treatment duration, climate types and N_2_-fixing or non-N_2_-fixing plants as moderator variables. To avoid confounding the responses of variables to experimental treatments, observations were categorized according to moderator variables in four categories: plant taxonomic group (angiosperm or gymnosperm), fertilizer type (including NH_4_NO_3_, Urea, ${\text{NH}}_{4}^{+}$-N, ${\text{NO}}_{3}^{-}$-N or Urea+ NH_4_NO_3_), N-addition level (N-addition ranged from 9 kg N ha^−1^ yr^−1^ to 600 kg N ha^−1^ yr^−1^, which were divided into low [<100 kg N ha^−1^ yr^−1^] and high levels [≥100 kg N ha^−1^ yr^−1^]) and treatment duration (experimental N addition treatment durations ranged from 4 weeks to 16 years, which were divided into short [<2 yr] and long [≥ 2 yr] terms).

### Meta-analysis

The natural log-transformed response ratio (*RR*), defined as the “effect size,” was used to measure the response of plant economic (foliage) and hydraulic traits to N addition (Hedges et al., 1999). For variables with negative values (water potential and *P*_50_), we used the inverse of effect size to avoid the miscalculation. The response ratio for each individual observation was calculated as the ratio of its mean value in the N fertilization treatment group (*X*_*t*_) to that in the control group (*X*_*c*_) (Equation 1), and then log transformed (*RR*) to conform with the statistical requirements of meta-analysis (Hedges et al., 1999):

(1) $$\begin{array}{r} \text{ln }RR=\text{ln }\left({\bar{X}}_{t}/{\bar{X}}_{c}\right)=\text{ln }\left({\bar{X}}_{t}\right)-\text{ln }\left({\bar{X}}_{c}\right) \end{array}$$

Note that this analysis involves a non-linear transformation of the data, therefore the response ratios for related physiological variables are not directly comparable. For example ln *RR* for *iWUE* cannot be directly compared to the ratio between the ln *RR* for net photosynthetic rate and the ln *RR* of stomatal conductance.

In addition, the mean, standard deviation $\left(SD=SE\sqrt{n}\right)$ and sample size of N-addition treatment (*S*_t_, *n*_t_) and control (*S*_c_, *n*_c_) group were used to calculate the variance of effect size (*v*) (Equation 2). The weighting factor (*w*) of each observation was calculated as the inverse of the variance (Equation 3).

(2) $$\begin{array}{r} v=\frac{S_{t}^{2}}{n_{t}{\bar{X}}_{t}^{2}}+\frac{S_{c}^{2}}{n_{c}{\bar{X}}_{c}^{2}} \end{array}$$

(3) $$\begin{array}{r} w=\frac{1}{v} \end{array}$$

Since some cases in our study had two or more observations, we adjusted the weight by the total number of observations per study to reduce the weight from the same site, and used the total weighting factor (*w*′) to calculate the mean effect size ($\bar{\text{ln }RR^{\prime }}$) (Bai et al., 2013; Li et al., 2015; Equations 4–6):

(4) $$\begin{array}{r} W^{\prime }=\text{w}/\text{n} \end{array}$$

(5) $$\begin{array}{r} \text{ln }RR^{\prime }=w^{\prime }\cdot \text{ln }RR \end{array}$$

(6) $$\begin{array}{c} \bar{\text{ln }RR^{\prime }}=\frac{\underset{i}{\sum }\text{ln }RR_{i}\prime }{\underset{i}{\sum }w_{i}^{\prime }} \end{array}$$

Where n is the total number of observations per study, ln*RR*′is weighted effect size, ln *RR*_*i*′_ and *w*_*i*′_ are ln*RR*′ and *w* of the *i*th observation, respectively.

A fixed effects model was used to determine whether N addition had a significant effect on each variable using Metawin 2.1 software. If the 95% CI of $\bar{\text{ln }RR^{\prime }}$ for a variable did not overlap with zero, the effect of N addition on the variable was considered to be significantly increased or significantly reduced (less negative or more negative for the variables with negative values); if not, the effects were considered to be not significant. Statistical analyses were performed using R 3.4.0. For a better explanation, the percentage change caused by simulated nitrogen deposition was transformed from the mean effect size:

(7) $$\begin{array}{r} \left(e^{\bar{\text{ln }RR^{\prime }}}-1\right)\times 100\% \end{array}$$

In addition, the heterogeneity within (*Q*_w_) and between (*Q*_b_) moderator levels was compared using mixed effects models to assess the significance of each categorical moderator (Borenstein et al., 2009). The heterogeneity analysis was applied using Metawin 2.1 software.

## Results

**Figure 1:**
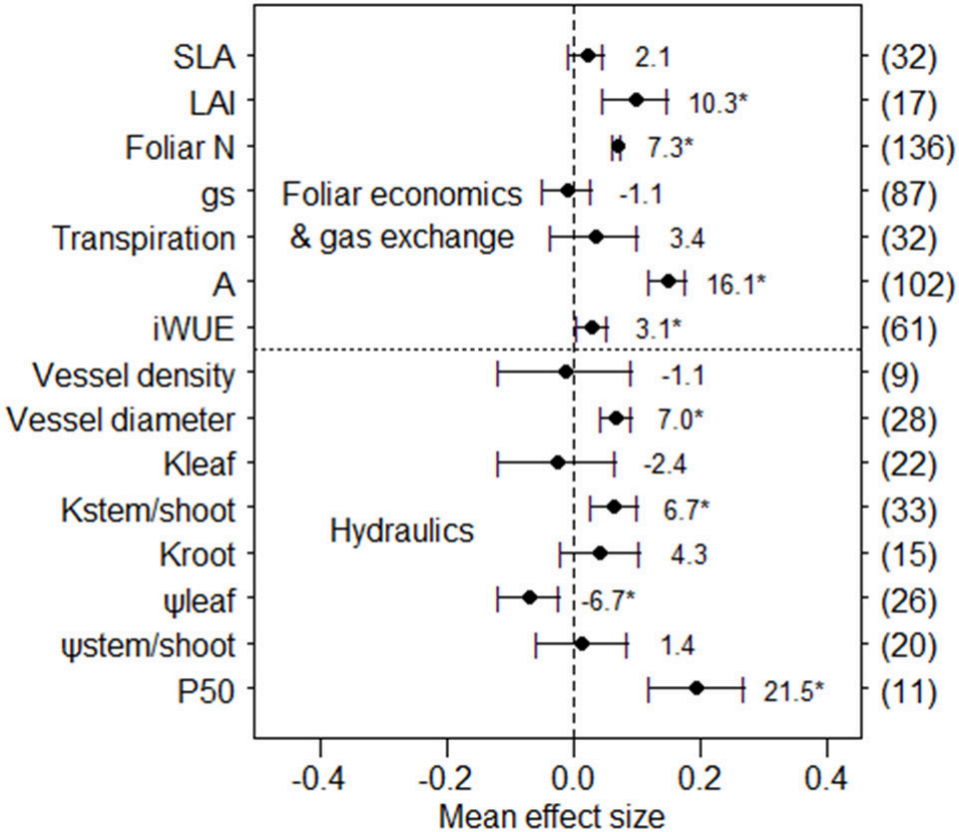
Mean effect sizes of N addition on two group variables. Error bars indicate 95% confidence intervals (CIs). The number in parentheses represents the sample size for each variables. The percentage of change (%) for each variable is shown next to the point. The “^*^” denotes a significant response. Data are shown for specific leaf area (SLA), leaf area index (LAI), foliar N content (foliar N), stomatal conductance (*g*_s_), Transpiration rate (Transpiration), net photosynthetic rate (A), intrinsic water-use efficiency (*iWUE*), hydraulic conductance of leaves (*K*_leaf_, stems/shoots (*K*_stem/shoot_) and roots (*K*_root_), leaf water potential (ψ_leaf_), stem/shoot water potential (ψ_stem/shoot_), and water potential corresponding to 50% loss of hydraulic conductivity (*P*_50_).

Considering the entire dataset of plant leaf economics and gas exchange, simulated nitrogen deposition significantly increased leaf area index (+10.3%), foliar N content (+7.3%), net photosynthetic rate (+16.1%) and intrinsic water-use efficiency (+3.1%, Figure 1), but did not change specific leaf area, stomatal conductance, and transpiration rate. For hydraulic traits, the observed numbers of vessel density, root hydraulic conductance, and *P*_50_ were less than 20. Based on the current limited number of observations, results indicated that N addition significantly increased vessel diameter (+7.0%), but did not affect vessel density. In addition, N fertilization significantly increased hydraulic conductance in stems/shoots (+6.7%), but not in leaves and roots. Water potentials were significantly reduced with nitrogen deposition in leaves (−6.7%) but not in stems/shoots, while *P*_50_ was significantly increased (+21.5%).

Because not all types of data were available for all studies, which studies and plants had available data for analyses varied for each functional trait we analyzed, as did sample size (Figure 1). Since these non-overlapping data could influence our conclusions about the relationships among functional traits, we checked for an overlap effect in our data for foliar N content, net photosynthetic rate, and *iWUE*. Analyzing only the data for studies that had both photosynthesis and foliar N content, we found that photosynthesis significantly increased 16.3% (*n* = 55) under N addition in the overlapped data (non-overlapped result was 16.1%, *n* = 102), and foliar N content significantly increased 13.9% (*n* = 55) in the overlapped data (non-overlapped result was 7.3%, *n* = 136). For overlapped data from studies that included both photosynthesis and *iWUE*, we found that photosynthesis significantly increased 10.6% (*n* = 45) and *iWUE* increased 3.1% (*n* = 45), but the effect on *iWUE* was not significant (non-overlapped results were +16.1%, *n* = 102, and +3.1%, *n* = 61, respectively). Overall, the effect of including non-overlapping data from different studies and species had a minimal influence on results for the functional traits we assessed.

**Figure 2:**
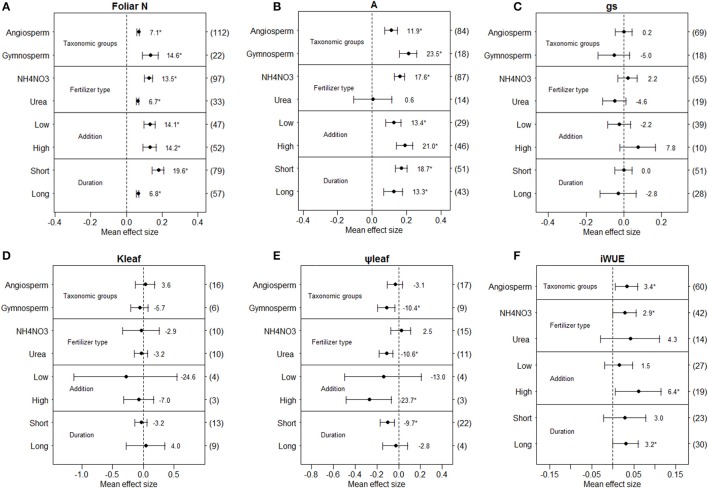
Mean effect sizes of N addition on foliar N content **(A)**, net photosynthetic rate **(B)**, stomatal conductance **(C)**, leaf hydraulic conductance **(D)**, leaf water potential **(E)**, and intrinsic water-use efficiency **(F)**. Error bars indicate 95% confidence intervals (CIs). The number in parentheses represents the sample size for each variables. The percentage of change (%) for each variable is shown next to the point. The variables are categorized into different groups according to plant taxonomic groups, fertilizer type, N-addition levels and treatment durations.

We found different responses to N deposition for angiosperms and gymnosperms (Figure 2). N addition significantly increased foliar N content and net photosynthetic rate in both angiosperms and gymnosperms (Figures 2A,B), but did not significantly affect stomatal conductance and leaf hydraulic conductance in either taxonomic group (Figures 2C,D). Leaf water potential (−10.4%, Figure 2E) was significantly reduced in gymnosperms, while *iWUE* (+3.4%) was significantly increased in angiosperms. The number of gymnosperm observations of *iWUE* was too small for meta-analysis (Figure 2F).

Overall, different fertilizer types induced different effects on the examined variables, although the number of observations from some fertilizer types (e.g. ${\text{NH}}_{4}^{+}$-N, ${\text{NO}}_{3}^{-}$-N, urea + NH_4_NO_3_) were too few for meta-analysis, limiting comparison to NH_4_NO_3_ and Urea (Figure 2). Based on the current limited number of observations, N addition had no significant effects on stomatal conductance and leaf hydraulic conductance in either NH_4_NO_3_ or urea addition (Figures 2C,D). Foliar N content increased with N addition in both NH_4_NO_3_ and urea addition, while net photosynthetic rate (+17.6%) and *iWUE* (+2.9%) were significantly increased only for NH_4_NO_3_ addition, leaf water potential was significantly decreased only for urea addition (−10.6%, Figure 2).

When N deposition was considered separately for low and high N-addition, we found few differences between levels of addition (Figure 2). Foliar N content and net photosynthetic rate were significantly increased in both low and high N-addition levels (Figures 2A,B), while leaf water potential (−23.7%, Figures 2E) and *iWUE* (+6.4%, Figure 2F) were significantly affected in high N-addition level only. Stomatal conductance and leaf hydraulic conductance were not significantly affected for either low or high N-addition levels (Figures 2C,D).

When the effect of simulated N deposition was analyzed separately by length of treatment, stomatal conductance and leaf hydraulic conductance were not significantly affected in either short-term or long-term experiments (Figures 2C,D). Both net photosynthetic rate and foliar N content were significantly increased in both short-term and long-term experiments, while leaf water potential (−9.7%) was significantly reduced in short-term experiments only, while the opposite occurred for *iWUE* (Figure 2).

Based on the current limited data (Table S2), there were no significantly different effects on foliar N content, net photosynthetic rate, stomatal conductance and leaf hydraulic conductance in different climate types. However, N addition did reduce leaf water potential in subtropical climate, but not in temperate climate, and intrinsic water-use efficiency significantly increased under N addition in temperate climate while not in subtropical and tropical climate. In addition, N addition significantly reduced net photosynthetic rate for N_2_-fixing plants while increased photosynthesis was found for non-N_2_-fixing plants, and intrinsic water-use efficiency significantly increased with for non-N_2_-fixing plants while not for N_2_-fixing plants.

## Discussion

### Effects of N addition on woody plant leaf economics and gas exchange

**Figure 3:**
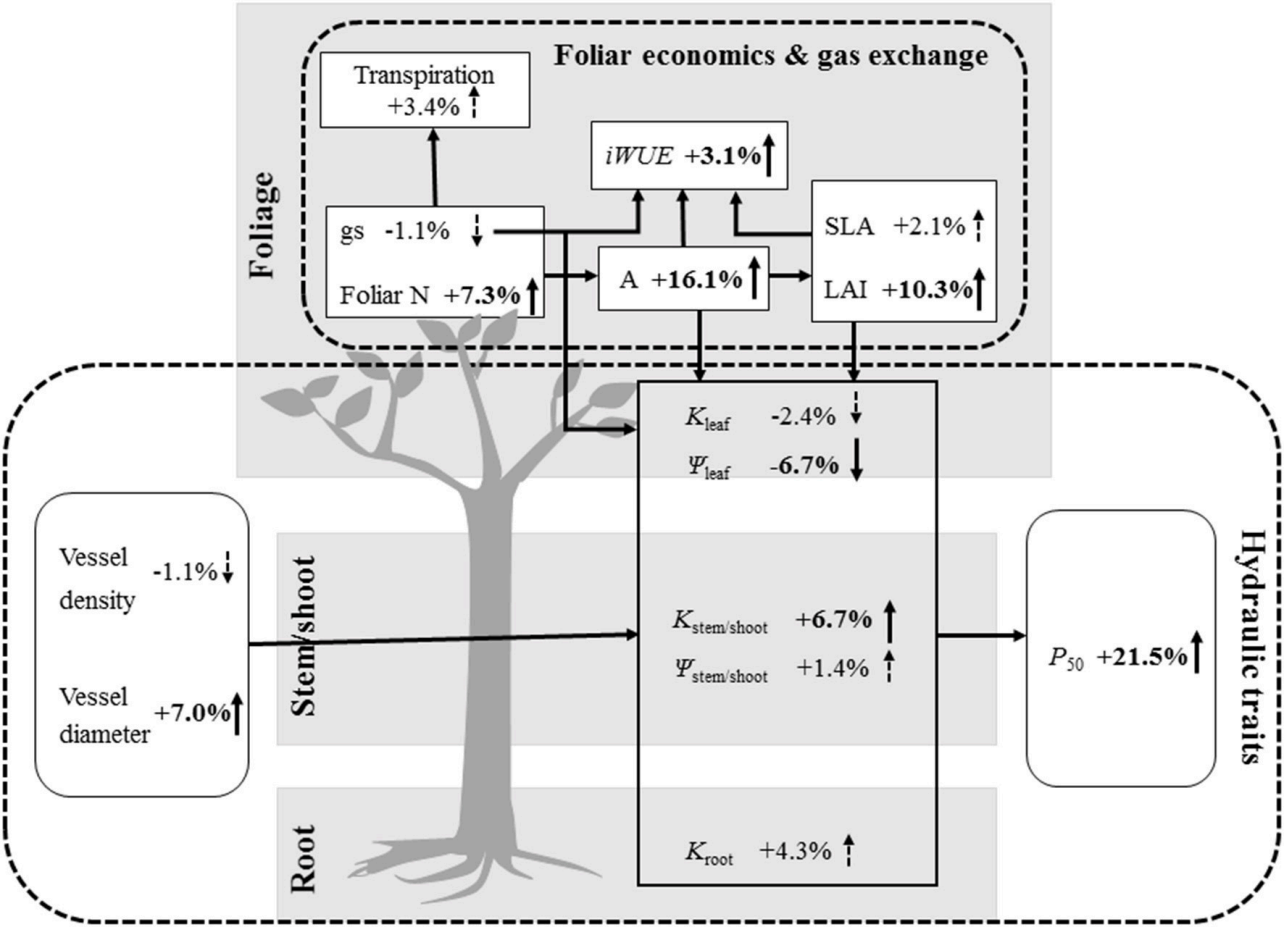
Responses of leaf economics, gas exchange, and hydraulic traits to N addition. Transpiration, Transpiration rate; gs, stomatal conductance; foliar N, foliar N content; *iWUE*, intrinsic water-use efficiency; A, net photosynthetic rate; SLA, specific leaf area; LAI, leaf area index; Kleaf, Kstem/shoot and Kroot, hydraulic conductance of leaves, stems/shoots and roots; ψleaf, leaf water potential; ψstem/shoot, stem/shoot water potential; *P*_50_, water potential corresponding to 50% loss of hydraulic conductivity. The numbers and arrows represent increases or decreases under N addition, respectively, and significant changes are indicated with a solid line, while dashed lines represent results that were not significant.

Stomatal closure reduces water loss from plants and concurrently lowers CO_2_ diffusion into the leaf, thereby playing a fundamental role in controlling plant transpiration and photosynthesis (Tardieu and Simonneau, 1998). Overall across plant taxonomic groups, our meta-analysis showed that N addition did not significantly affect stomatal conductance and transpiration rate, but increased net photosynthetic rate (+16.1%) and foliar N content (+7.3%, Figure 1). This result illustrates the strong link between increased production of photosynthetic proteins (e.g., Rubisco) and enhanced C assimilation rate (Evans, 1989; Reich et al., 1998), which is consistent with many previous findings that N-addition increases photosynthetic performance (Hirose and Werger, 1987; Chen et al., 1993; Shiratsuchi et al., 2006). This enhancement of net photosynthetic rate combined with increased leaf area index (+10.3%) we observed under N fertilization may lead to increased woody plant primary production. Moreover, N accumulation not only increased foliar N content (Bubier et al., 2011) but also enhanced pant nutritional status to promote growth (Marques et al., 1983; Aber et al., 2003; Ågren, 2004; Aoyama et al., 2014; Montti et al., 2014). Increased net photosynthetic rate and unchanged stomatal conductance under N addition in our meta-analysis was consistent with the effect of increased *iWUE* (+3.1%), which is the ratio between these two parameters (Boyer, 1996). Brueck (2008) also reported that N supply promotes *iWUE*. However, the response of *iWUE* to increasing N availability can vary depending on leaf age, leaf morphology (e.g., specific leaf area) and plant environmental conditions (e.g., light, Brueck, 2008). In our meta-analysis, we did not find a significant response of specific leaf area to N addition, suggesting that changes in specific leaf area are not responsible for the increase in *iWUE* we observed (Figure 3).

Leaves impose significant constraints on plant maximum stomatal conductance and photosynthetic capacity (Brodribb et al., 2005), and leaves are more hydraulically vulnerable than stems or roots (Salleo et al., 2001; Brodribb and Holbrook, 2003; Hao et al., 2008, 2013). Thus, leaf functional traits are considered critical factors influencing plant hydraulics. In our meta-analysis, unchanged specific leaf area with N fertilization suggests that plants did not increase their investments in leaf density and thickness, suggesting N addition will not alter leaf lifespan, the proportion of lignified tissue, leaf toughness, or structural defenses against herbivory (Wright et al., 2004; Poorter et al., 2009; Prado et al., 2014; Reich, 2014). Conversely, N addition increased leaf area index, nutrient content (N) and net photosynthetic rate, which is consistent with the principal trade-off between mass investment in leaf longevity and in new leaf production (Pérez-Ramos et al., 2013; Figure 3).

N addition induced a greater positive effect on net photosynthetic rate in gymnosperms than in angiosperms (*P* = 0.027, Table S1, Figure 2B). The different response to N addition between gymnosperms and angiosperms was presumably related to higher specific leaf area in gymnosperms, which could lead to different effects on primary production between gymnosperms and angiosperms (Li et al., 2017). For *iWUE*, the small number of observations on gymnosperms under N addition limited the power of our meta-analysis (*n* = 1, Figure 2F), but the increased *iWUE* in angiosperms with N-addition was consistent with overall changes in *iWUE*. This result is similar to that of Leonardi et al. (2012), who reported that N deposition induced a positive effect on *iWUE* in both angiosperms and gymnosperms.

Urea and NH_4_NO_3_ were the most frequently used fertilizers, and the small number of observations for ${\text{NH}}_{4}^{+}$-N, ${\text{NO}}_{3}^{-}$-N or Urea + NH_4_NO_3_ addition limited the power of our meta-analysis (Figure 2). In our results, urea and NH_4_NO_3_ addition did not induce different effects on stomatal conductance (Figure 2C), while only NH_4_NO_3_ addition induced significantly positive effects on net photosynthetic rate and *iWUE* (Figures 2B,F). This could be due to nitrate from NH_4_NO_3_ which could alter plant N uptake kinetics (Piwpuan et al., 2013) and promote plant cation absorption, but concurrently inhibit phosphorus absorption (Ruan et al., 2000). Alternately, previous studies suggested that high ${\text{NH}}_{4}^{+}$ addition can reduce plant growth and survival rate (Koerselman et al., 1990; Pearce et al., 2003) due to the potential toxicity of ${\text{NH}}_{4}^{+}$ to vascular plants (Paulissen et al., 2004), while urea could be rapidly hydrolyzed by the enzyme urease and predominantly released as ${\text{NH}}_{4}^{+}$ (Singh et al., 2013), thereby impairing the facilitation effect of N fertilization on net photosynthetic rate. Consistent with this, mixed ${\text{NO}}_{3}^{-}$ and ${\text{NH}}_{4}^{+}$ addition produced greater effects on foliar N and photosynthesis than urea (Liu et al., 2016).

Our finding that *iWUE* increased with high levels of N-addition but not with low levels is consistent with the previous synthesis results by Leonardi et al. (2012), who demonstrated a significant association of N deposition rate with *iWUE*, although the extent of increased *iWUE* was mainly controlled by the ratio between leaf intercellular and ambient CO_2_ concentrations. Similar effects on foliar N content at low and high levels of N addition could have occurred because N concentration in leaves was saturated at low levels (Wilson and Tiley, 1998; Pardo et al., 2007). Timing of N addition also affected results, as increases in both net photosynthetic rate and foliar N content with N addition were more pronounced in short-term experiments (*P* < 0.05, Table S1), while *iWUE* only increased in long-term experiments (Figure 2F). Different trends in net photosynthetic rate and *iWUE* response to N addition with increasing duration of N addition would be consistent with long-term N accumulation altering plant C allocation patterns and metabolic processes (Cramer and Lewis, 1993; McCarthy et al., 2006). A reduced response in foliar N uptake from short-term to long-term treatments suggests that plants acclimate to N addition. N addition promoted *iWUE* in temperate climate but not in subtropical and tropical climates (Table S2) suggesting that plants have lower *iWUE* under climate conditions with higher temperatures and precipitation. It is also interesting to find that photosynthesis was significantly reduced under N addition for N_2_-fixing plants but significantly increased for non-N_2_-fixing plants (Table S2), this result induced no significantly effects on *iWUE*, though the data was limited. A previous meta-analysis reported that N_2_-fixing plants had higher protein content compared to non-N_2_-fixing plants under N addition treatment (Liu et al., 2016), thus instead of promoting, N addition might become a burden for photosynthesis in N_2_-fixing plants.

### Effects of N addition on woody plant hydraulics

The response of vessel hydraulic architecture to N addition can reveal how plants regulate their conducting system under variable plant-nutrient conditions. We found that N fertilization significantly increased vessel diameter, but did not change vessel density (Figure 1), which increases hydraulic conductivity (Hacke et al., 2009; Pittermann et al., 2011) and the susceptibility to drought-induced hydraulic failure (Harvey and Van Den Driessche, 1999; McElrone et al., 2004; Wheeler et al., 2005; Villar-Salvador et al., 2013; but see Gleason et al., 2016). This is inconsistent with Borghetti et al. (2017) who reported that N deposition provided capacity to withstand the risks of embolism due to higher vessel density. We found that N addition increased hydraulic conductance in stems/shoots, but not in leaves or roots (Figures 1, 3). Significantly increased hydraulic conductance in the stem or shoot under N addition with a concomitant increase in LAI may have a positive effect on the whole-plant hydraulic conductance. However, leaves and roots have been demonstrated to be two major bottlenecks in whole-plant water flow (Tyree et al., 1998; Nardini and Tyree, 1999; Sack et al., 2003, 2005). The lack of significant effects on leaf hydraulic conductance in response to N addition was presumably because N fertilization stimulated plant leaf production (increased LAI in our meta-analysis), but impaired hydraulic conductance in leaves, which in turn counteracted the facilitation effects of N addition on leaf hydraulic conductance. Thus, more studies including whole-plant hydraulic conductance are needed to understand the effects of N deposition on plant hydraulic conductance. In roots, hydraulic resistance is typically attributed to high radial resistance in fine roots before water reaches the xylem conduits (Tsuda and Tyree, 1997), hence larger fine root surface area would strongly increase water absorption and thus enhance root hydraulic conductance. A previous meta-analysis demonstrated that N addition did not alter fine root diameter or length, but did decrease fine root quantity (Li et al., 2015), implying that fine root surface area for water absorption was reduced. This effect could explain the lack of an effect on root hydraulic resistance that we found.

Generally, the distal portions of plants should be more vulnerable to embolism than stems, which could protect the more C-costly parts (e.g., stems; hydraulic segmentation hypothesis; Hao et al., 2008; Bucci et al., 2013; Scholz et al., 2014; Johnson et al., 2016; Pivovaroff et al., 2016). Therefore, it is logical that reduced water potential and increased embolism occurs first in distal plant parts. In our meta-analysis, we found that N addition reduced water potential in leaves, but not in stems/shoots, which could increase the risk of embolism in leaves under unfavorable water conditions (Johnson et al., 2009). Despite the decrease in water potential in leaves under N addition, stomatal conductance was not affected, indicating a reduced sensitivity of stomatal conductance to water stress. Since no water potential data were available for our meta-analysis for roots, another important distal plant tissue, more studies should be carried out to explore the effect of N fertilization on root water potential and to understand this phenomenon better. In addition, increased *P*_50_ with N addition suggests that N fertilization increased xylem vulnerability to cavitation, which could reduce overall plant tolerance to environmental stress (e.g., drought, freeze-thaw cycles). Combined with increased hydraulic conductance in stems/shoots, these results suggest that xylem safety was traded for xylem hydraulic efficiency (safety-efficiency hypothesis; Gleason et al., 2016), which would be disadvantageous under unfavorable water conditions (Figure 3).

Overall, across 61 woody plant species, the response of leaf hydraulic conductance to N addition was not affected by moderator variables (taxonomic groups, fertilizer types, N-addition levels and treatment durations, Figure 2D). Decreased leaf water potential with N addition under urea addition, high N addition and short N fertilization terms (Figure 2E) indicates that fertilized plants were more likely to risk embolism in leaves than unfertilized plants, a risk that could increase during drought. However, since plants replace conducting area via new growth (and perhaps refill embolism) more readily under high N availability (McDowell et al., 2011; Zeppel et al., 2015), plant resilience to water deficit may vary depending on the timing and duration of drought. Hence, more long-term manipulation experiments with interactions of N addition and drought are needed to reveal plant tolerances to environmental changes. It should be noted that the number of observations for some groups or levels of moderating variables for both leaf hydraulic conductance and water potential were small (*n* < 10, Figures 2D,E), which limited the power of our meta-analysis.

## Conclusions

We found that N addition increased foliar N content, leaf area index, net photosynthetic rate, and *iWUE* across a variety of woody plant species, which may enhance plant primary production and N demand. For plant hydraulic traits, N addition increased hydraulic conductance in stems/shoots, due to greater vessel diameter with N addition, which we observed in our study. Increased vessel diameter and no change in vessel density suggested that N addition elevated the risk of embolism and hydraulic dysfunction, as reflected in the effects of N addition on *P*_50_, a tradeoff between xylem safety and efficiency. Moreover, N addition significantly reduced leaf water potential but not leaf hydraulic conductance, which indicated that leaves were more likely to experience embolism in under stress than stems or shoots. These results also confirmed the tradeoff between C and hydraulic economics because N addition increased plant C uptake, through its effects on net photosynthetic rate, *iWUE*, and leaf area index, but also makes plants more vulnerable to hydraulic dysfunction. We present the results of different tree organs of hydraulic conductance, and argue that it will be essential to study the whole-plant hydraulic conductance under N deposition. In order to accurately predict plant performance and dynamics under climate change, further studies should focus on the effects of multiple stressors (drought and N fertilization) and their interactions. In the future, understanding the trade-off between the leaf economics of C and water exchange could improve prediction of hydraulic dysfunction need to anticipate threshold events in vegetation change that may come to dominate certain ecosystem response to multiple global change effects. This meta-analysis offers a comprehensive perspective of N addition effects on foliar economics, gas exchange, and hydraulic traits, and exposes several knowledge gaps in fertilizer usage, and responses under multiple environmental stresses.

## Author contributions

All authors contributed to the development of ideas, and interpretation of results. HZ, FY, and WL: collected raw data and performed statistical analyses; HZ, DG, WL, and HA: co-wrote the manuscript and all authors contributed to revisions.

### Conflict of interest statement

The authors declare that the research was conducted in the absence of any commercial or financial relationships that could be construed as a potential conflict of interest.

## Supplementary material

Data Sheet 1 The list of 56 papers used in the meta-analysis.

Table S1 Summary of the heterogeneity between moderator levels (Qb) (plant taxonomic groups, fertilizer types, N-addition levels and treatment durations) and *P*-value.

Table S2 The percentage change (%) caused by N addition for each variable and the variables are categorized into different groups according to climate type and N2-fixing or non-N2-fixing plants.
