# Supplementary material for: Responses of Woody Plant Functional Traits to Nitrogen Addition: A Meta-Analysis of Leaf Economics, Gas Exchange, and Hydraulic Traits
Source: Front Plant Sci. 2018 May 23;9:683. doi: 10.3389/fpls.2018.00683 (PMC5974508; doi:10.3389/fpls.2018.00683)
Supplement: Data Sheet 1 — The list of 56 papers used in the meta-analysis. [file Data_Sheet_1.PDF]

List all of 56 papers used in the meta-analysis

Bubier JL, Smith R, Juutinen S, Moore TR, Minocha R, Long S, Minocha S (2011) Effects of nutrient addition on leaf chemistry, morphology, and photosynthetic capacity of three bog shrubs. Oecologia. 167:355-368.

Chandler JW, Dale JE (1995) Nitrogen deficiency and fertilization effects on needle growth and Photosynthesis in Sitka spruce (*picea sitchensis*). Tree Physiol. 15:813-817.

Chen LH, Dong TF, Duan BL (2014) Sex-specific carbon and nitrogen partitioning under N deposition in Populus cathayana. Trees-Struct Funct. 28:793-806.

DesRochers A, van den Driessche R, Thomas BR (2006) NPK fertilization at planting of three hybrid poplar clones in the boreal region of Alberta. For Ecol Manage. 232:216-225.

Ding WJ, Wang RQ, Yuan YF, Liang XQ, Liu J (2012) Effects of nitrogen deposition on growth and relationship of Robinia pseudoacacia and Quercus acutissima seedlings. Dendrobiology. 67:3-13.

Domec JC, Palmroth S, Ward E, Maier CA, Therezien M, Oren R (2009) Acclimation of leaf hydraulic conductance and stomatal conductance of Pinus taeda (*loblolly pine*) to long-term growth in elevated CO2 (free-air CO2 enrichment) and N-fertilization. Plant Cell Environ. 32:1500-1512.

Du EZ, Fang JY (2014) Weak growth response to nitrogen deposition in an old-growth boreal forest. Ecosphere. 5:1-9.

Eller F, Jensen K, Reisdorff C (2017) Nighttime stomatal conductance differs with nutrient availability in two temperate floodplain tree species. Tree Physiol. 37:428-440.

Faustino LI, Bulfe NML, Pinazo MA, Monteoliva SE, Graciano C (2013) Dry weight partitioning and hydraulic traits in young Pinus taeda trees fertilized with nitrogen and phosphorus in a subtropical area. Tree Physiol. 33:241-251.

Faustino LI, Moretti AP, Graciano C (2015) Fertilization with urea, ammonium and nitrate produce different effects on growth, hydraulic traits and drought tolerance in Pinus taeda seedlings. Tree Physiol. 35:1062-1074.

Forrester DI, Collopy JJ, Beadle CL, Warren CR, Baker TG (2012) Effect of thinning, pruning and nitrogen fertiliser application on transpiration, photosynthesis and water-use efficiency in a young Eucalyptus nitens plantation. For Ecol Manage. 266:286-300.

Fusaro L, Salvatori E, Manes F (2017) Effects of nitrogen deposition, drought and their interaction, on functional and structural traits of *Fraxinus ornus* L. and *Quercus ilex* L. Plant Biosyst. 151:174-189.

Goodman RC, Oliet JA, Sloan JL, Jacobs DF (2014) Nitrogen fertilization of black walnut (*Juglans nigra* L.) during plantation establishment. Physiology of production. Eur J Forest Res. 133:153-164.

Graciano C, Guiamet JJ, Goya JF (2005) Impact of nitrogen and phosphorus fertilization on drought responses in Eucalyptus grandis seedlings. For Ecol Manage. 212:40-49.

Guo X, Wang RQ, Chang RY, Liang XQ, Wang CD, Luo YJ, Yuan YF, Guo WH (2014) Effects of nitrogen addition on growth and photosynthetic characteristics of Acer truncatum seedlings. Dendrobiology. 72:151-161.

Harrington RA, Fownes JH, Vitousek PM (2001) Production and resource use efficiencies in N- and P-limited tropical forests: A comparison of responses to long-term fertilization. Ecosystems. 4:646-657.

Harvey HP, Van DDR (1999) Nitrogen and potassium effects on xylem cavitation and water-use

efficiency in poplars. Tree Physiol. 19:943-950.

Hattenschwiler S, Korner C (1998) Biomass allocation and canopy development in spruce model ecosystems under elevated CO2 and increased N deposition. Oecologia. 113:104-114.

Jennings KA, Guerrieri R, Vadeboncoeur MA, Asbjornsen H (2016) Response of Quercus velutina growth and water use efficiency to climate variability and nitrogen fertilization in a temperate deciduous forest in the northeastern USA. Tree Physiol. 36:428-43.

Jing H, Zhou H, Wang G, Xue S, Liu G, Duan M (2017) Nitrogen Addition Changes the Stoichiometry and Growth Rate of Different Organs in Pinus tabuliformis Seedlings. Front Plant Sci. 8:1-10.

Jing YL, Guan DX, Wu JB, Wang AZ, Jin CJ, Yuan FH (2016) Photosynthate supply drives soil respiration of Fraxinus mandshurica seedlings in northeastern China: evidences from a shading and nitrogen addition experiment. J Forestry Res. 27:1271-1276.

Li Y, Zhang XL, Yang YQ, Duan BL (2013) Soil cadmium toxicity and nitrogen deposition differently affect growth and physiology in Toxicodendron vernicifluum seedlings. Acta Physiol Plant. 35:529-540.

Li YY, Liu JX, Chen GY, Zhou GY, Huang WJ, Yin GC, Zhang DQ, Li YL (2015) Water-use efficiency of four native trees under CO2 enrichment and N addition in subtropical model forest ecosystems. J Plant Ecol. 8:411-419.

Liu JX, Zhang DQ, Zhou GY, Duan HL (2012) Changes in leaf nutrient traits and photosynthesis of four tree species: effects of elevated CO2 , N fertilization and canopy positions. J Plant Ecol. 5:376-390.

Lovelock CE, Ball MC, Choat B, Engelbrecht BMJ, Holbrook NM, Feller IC (2006a) Linking physiological processes with mangrove forest structure: phosphorus deficiency limits canopy development, hydraulic conductivity and photosynthetic carbon gain in dwarf Rhizophora mangle. Plant Cell Environ. 29:793-802.

Lovelock CE, Feller IC, Ball MC, Engelbrecht BMJ, Ewe ML (2006b) Differences in plant function in phosphorus- and nitrogen-limited mangrove ecosystems. New Phytol. 172:514-522.

Lovelock CE, Feller IC, Mckee KL, Engelbrecht BMJ, Ball MC (2004) The Effect of Nutrient Enrichment on Growth, Photosynthesis and Hydraulic Conductance of Dwarf Mangroves in Panamá Funct Ecol. 18:25–33.

Mao QZ, Watanabe M, Imori M, Kim YS, Kita K, Koike T (2012) Photosynthesis and nitrogen allocation in needles in the sun and shade crowns of hybrid larch saplings: effect of nitrogen application. Photosynthetica. 50:422-428.

Marshall JD, Dawson TE, Ehleringer JR (1994) Integrated nitrogen, carbon and water relations of a xylem- tapping mistletoe following nitrogen- fertilization of the host. Oecologia. 100:430-438.

Martin KC, Bruhn D, Lovelock CE, Feller IC, Evans JR, Ball MC (2010) Nitrogen fertilization enhances water-use efficiency in a saline environment. Plant Cell Environ. 33:344-357.

Marzuoli R, Monga R, Finco A, Gerosa G (2016) Biomass and physiological responses of Quercus robur (L.) young trees during 2 years of treatments with different levels of ozone and nitrogen wet deposition. Trees-Struct Funct. 30:1995-2010.

Mo JM, Li DJ, Gundersen P (2008) Seedling growth response of two tropical tree species to nitrogen deposition in southern China. Eur J Forest Res. 127:275-283.

Nagakura J, Kaneko S, Takahashi M, Tange T (2008) Nitrogen promotes water consumption in seedlings of Cryptomeria japonica but not in Chamaecyparis obtusa. For Ecol Manage. 255:2533-2541.

Palmroth S, Bach LH, Nordin A, Palmqvist K (2014) Nitrogen-addition effects on leaf traits and

photosynthetic carbon gain of boreal forest understory shrubs. Oecologia. 175:457-470.

Pascual M, Villar JM, Rufat J (2016) Water use efficiency in peach trees over a four-years experiment on the effects of irrigation and nitrogen application. Agric Water Manage. 164:253-266.

Pivovaroff AL, Santiago LS, Vourlitis GL, Grantz DA, Allen MF (2016) Plant hydraulic responses to long-term dry season nitrogen deposition alter drought tolerance in a Mediterranean-type ecosystem. Oecologia. 181:721-731.

Plavcová L, Hacke UG, Almeida-Rodriguez AM, Eryang LI, Douglas CJ (2013) Gene expression patterns underlying changes in xylem structure and function in response to increased nitrogen availability in hybrid poplar. Plant Cell Environ. 36:186-199.

Plavcova L, Hacke UG (2012) Phenotypic and developmental plasticity of xylem in hybrid poplar saplings subjected to experimental drought, nitrogen fertilization, and shading. J Exp Bot. 63:6481-6491.

Spannl S, Homeier J, Bräuning A (2016) Nutrient-induced modifications of wood anatomical traits of *Alchornea lojaensis* (Euphorbiaceae). Frontiers in Earth Science. 4: 2296-6463.

Tripathi SN, Raghubanshi AS (2014) Seedling growth of five tropical dry forest tree species in relation to light and nitrogen gradients. J Plant Ecol. 7:250-263.

Hacke UG, Plavcová L, Almeida-Rodriguez A, King-Jones S, Zhou W, Cooke JEK (2010) Influence of nitrogen fertilization on xylem traits and aquaporin expression in stems of hybrid poplar. Tree Physiol. 30:1016-1025.

Villar-Salvador P, Penuelas JL, Jacobs DF (2013) Nitrogen nutrition and drought hardening exert opposite effects on the stress tolerance of Pinus pinea L. seedlings. Tree Physiol. 33:221-32.

Wang AY, Wang M, Yang D, Song J, Zhang WW, Han SJ, Hao GY (2016) Responses of hydraulics at the whole-plant level to simulated nitrogen deposition of different levels in Fraxinus mandshurica. Tree Physiol. 36:1045-55.

Wang G, Liu F, Xue S (2017) Nitrogen addition enhanced water uptake by affecting fine root morphology and coarse root anatomy of Chinese pine seedlings. Plant Soil. 418:177-189.

Wang GL, Hu F (2014) Carbon allocation of Chinese pine seedlings along a nitrogen addition gradient. For Ecol Manage. 334:114-121.

Wu FZ, Bao WK, Li FL, Wu N (2008a) Effects of drought stress and N supply on the growth, biomass partitioning and water-use efficiency of Sophora davidii seedlings. Environ Exp Bot. 63:248-255.

Wu FZ, Bao WK, Li FL, Wu N (2008b) Effects of water stress and nitrogen supply on leaf gas exchange and fluorescence parameters of Sophora davidii seedlings. Photosynthetica. 46:40-48.

Wu FZ, Bao WK, Zhou ZQ, Wu N (2009) Carbon accumulation, nitrogen and phosphorus use efficiency of Sophora davidii seedlings in response to nitrogen supply and water stress. J Arid Environ. 73:1067-1073.

Wuyts K, Adriaenssens S, Staelens J, Wuytack T, Van Wittenberghe S, Boeckx P, Samson R, Verheyen K (2015) Contributing factors in foliar uptake of dissolved inorganic nitrogen at leaf level. Sci Total Environ. 505:992-1002.

Xu NN, Guo WH, Liu J, Du N, Wang RQ (2015) Increased nitrogen deposition alleviated the adverse effects of drought stress on Quercus variabilis and Quercus mongolica seedlings. Acta Physiol Plant. 37:107-118.

Yahdjian L, Gherardi L, Sala OE (2014) Grasses have larger response than shrubs to increased nitrogen availability: A fertilization experiment in the Patagonian steppe. J Arid Environ. 102:17-20.

Yang Y, Guo JY, Wang GX, Yang LD, Yang Y (2012) Effects of drought and nitrogen addition on photosynthetic characteristics and resource allocation of Abies fabri seedlings in eastern Tibetan Plateau. New Forests. 43:505-518.

Yao XQ, Liu Q (2009) Photosynthetic and physiological responses of *Swida hemsleyi* (C.K. schneid. Et wangerin) subjected to enhanced UV-B enhanced and to nitrogen suppy. Polish J Ecol. 57:483-494.

Zhang ZL, Liu GD, Zhang FC, Zheng CX, Ni FQ, Kang YH, Zeng Y (2014) Effects of nitrogen content on growth and hydraulic characteristics of peach (Prunus persica L.) seedlings under different soil moisture conditions. J Forestry Res. 25:365-375.

Zhao CZ, Liu Q (2009) Growth and physiological responses of Picea asperata seedlings to elevated temperature and to nitrogen fertilization. Acta Physiol Plant. 31:163-173.

Zhao CZ, Liu Q (2012) Effects of soil warming and nitrogen fertilization on leaf physiology of Pinus tabulaeformis seedlings. Acta Physiol Plant. 34:1837-1846.

Table S1 Summary of the heterogeneity between moderator levels (Q_b) (plant taxonomic groups, fertilizer types, N-addition levels and treatment durations) and *P*-value. Foliar N, foliar N content; A, net photosynthetic rate; Gs, stomatal conductance; K_{leaf}, leaf hydraulic conductance; ψ_{leaf}, leaf water potential; *iWUE*, intrinsic water-use efficiency.

		**Foliar N**	**A**	**Gs**	$\mathbf{K_{leaf}}$	$\mathbf{\psi_{leaf}}$	***iWUE***
**Angiosperm vs. Gymnosperm**	Q_b	11.100	11.074	1.348	1.070	2.730	-
	p	0.453	0.027	0.435	0.414	0.392	-
**NH_4NO_3 vs. Urea**	Q_b	33.266	8.510	4.368	0.0003	7.108	0.552
	p	0.156	0.060	0.381	0.988	0.146	0.587
**Low vs. High**	Q_b	29.187	5.289	7.794	3.500	24.611	2.521
	p	0.524	0.422	0.314	0.297	0.002	0.081
**Short vs. Long**	Q_b	43.154	16.020	1.031	0.253	2.452	0.464
	p	0.011	0.030	0.786	0.709	0.324	0.651

Table S2 The percentage change (%) caused by N addition for foliar N content (Foliar N), net photosynthetic rate (A), stomatal conductance (gs), leaf hydraulic conductance (K_{leaf}) , leaf water potential (ψ_{leaf}), and intrinsic water-use efficiency (*iWUE*). The variables are categorized into different groups according to climate type (temperate, subtropical and tropical) and N_2-fixing or non-N_2-fixing plants. Only the number of observations (n ≥10) were listed. The '*' denotes a significant response. The number in parentheses repres ents the sample size for each variables.

	Climate type			N_2-fixing or non-N_2-fixing plants	
	Temperate	Subtropical	Tropical	N_2-fixers	Non-N_2-fixers
**Foliar N**	13.2* (46)	18.8* (22)	6.6* (16)	14.7* (23)	7.1* (105)
**A**	6.0* (30)	17.3* (35)	16.0 (40)	-16.3* (16)	17.1 (86)
**gs**	-3.5 (48)	1.3 (34)	-2.1(5)	-4.8 (12)	-0.1 (69)
K_{leaf}	-2.9 (10)	-4.5 (10)	33 (2)	-0.3 (2)	-2.9 (20)
ψ_{leaf}	-3.6 (11)	-7.5* (15)	-	-	-6.7* (26)
iWUE	3.1* (33)	3.2 (20)	-6.2(8)	3.3 (16)	3.0* (45)
